# Calcium Plays a Double-Edged Role in Modulating Cadmium Uptake and Translocation in Rice

**DOI:** 10.3390/ijms21218058

**Published:** 2020-10-29

**Authors:** Shuo Zhang, Qi Li, Muhammad Mudassir Nazir, Shafaqat Ali, Younan Ouyang, Shuzhen Ye, Fanrong Zeng

**Affiliations:** 1 Collaborative Innovation Centre for Grain Industry, College of Agriculture, Yangtze University, Jingzhou 434025, China; q563850174@zju.edu.cn; 2 Institute of Crop Science, Zhejiang University, Hangzhou 310058, China; qili0605@zju.edu.cn (Q.L.); 11816109@zju.edu.cn (M.M.N.); 3 Department of Environmental Sciences and Engineering, GC University Faisalabad, Faisalabad 38000, Pakistan; 4 Department of Biological Sciences and Technology, China Medical University, Taichung 40402, Taiwan; 5 China National Rice Research Institute, Hangzhou 310006, China; ouyangyounan@caas.cn (Y.O.); shuzhen.ye@163.com (S.Y.)

**Keywords:** cadmium, calcium, crop safety, membrane transport, net Cd^2+^ influx, root-to-shoot translocation

## Abstract

Cadmium (Cd) contamination in soils poses great risks to both agricultural production and human health. Calcium (Ca) is an essential element playing a significant role in protecting plants against Cd toxicity. However, how Ca affects Cd uptake and translocation in rice is still not fully elucidated. In this study, the regulatory role of Ca in Cd uptake and upward translocation was investigated in rice at different growth stages. Our results showed that the supplement of 5 mM Ca significantly reduced Cd uptake by rice roots, because of their competition for Ca-permeable channels as an absorption site and Ca-induced downregulation of *OsNRAMP1* and *OsNRAMP5*. However, Ca application facilitated the upward translocation of Cd by both upregulating *OsHMA2* to induce xylem loading of Cd and downregulating *OsHMA3* to reduce vacuolar sequestration of Cd. Such contrary results suggested a double-edged role of Ca in regulating root Cd uptake and root-to-shoot Cd translocation in rice. Although it increased Cd content in the aboveground vegetative tissues during the whole growth period, the addition of 5 mM Ca eventually decreased Cd content in rice grains at the ripening stage. All these results suggest that Ca-based amendments possess great potential for the production of low-Cd rice grains.

## 1. Introduction

Cadmium (Cd) contamination has become one of the most severe environmental issues worldwide [1]. It has been estimated that about 30,000 tons of Cd pollutants are annually discharged into the environment [2]. Cd in soil is readily taken up and accumulated in plants, causing a number of detrimental impacts on plants, such as inhibiting plant growth and development, reducing photosynthesis and respiration, disturbing nutrient uptake and water relations, damaging cell membrane permeability, and disrupting the cellular redox homeostasis, consequently resulting in yield reduction and even plant death [3,4,5]. To make the matter worse, Cd accumulation in plant tissues, especially in the edible parts such as grains of cereal crops [6], can enter the food chain and, thus, pose a great threat to human health [4]. Rice (*Oryza sativa* L.) is a major component of the diet for more than three billion people, with almost 90% of rice production and consumption being reported in Asia [7]. However, the production of rice is now suffering a severe threat in the form of Cd pollution, and Cd-contaminated rice is one of the main sources of Cd exposure to humans [8,9,10]. Therefore, it is imperative to develop effective strategies to reduce Cd accumulation in rice for minimizing Cd intake into the human body.

In general, Cd accumulation in rice grains is mediated by four major processes: (i) Cd uptake by roots from soil, (ii) xylem-loading-mediated Cd translocation from root to shoot, (iii) redirection of transport through intervascular transfer at nodes, and (iv) Cd accumulation in grains through phloem. The molecular mechanisms conferring these four processes have been extensively investigated and reviewed [1,3,11]. As a nonessential and toxic element for plants, Cd is assumed to enter plant cells through the transporters for essential elements such as Ca, Fe, Zn, and Mn, due to their similarity in chemical and physical properties [12]. In rice, a series of transporters, such as natural resistance-associated macrophage proteins (OsNRAMP1, OsNRAMP5), iron-regulated transporter 1 (OsIRT1), and OsCd1[a major facilitator superfamily (MFS) protein], have been demonstrated to take up Cd in root cells [13,14,15,16]. Of them, OsNRAMP5 is considered as a major route of Cd uptake in rice root [15]. After root uptake, Cd is translocated from root to shoot through xylem loading, which is a key factor for Cd accumulation in aboveground tissues of rice [17]. OsHMA2 and OsHMA3 have been demonstrated to play an important role in this process, with OsHMA3 taking effect in Cd compartmentation into vacuoles in root cells [18] and OsHMA2 being identified as a major contributor to xylem loading [19]. After transfer from the xylem to phloem at nodes, Cd is favorably transported to the upper nodes and eventually into grains through phloem with the contribution of the node-localized Cd transporter OsLCT1 [8]. In addition, Cd ions (Cd^2+^) can also be passively transported through channel proteins transporting Ca^2+^, as they share similarities in terms of their charge and ionic radius. Many Ca-permeable channels, including depolarization-activated calcium channels (DACCs), hyperpolarization-activated calcium channels (HACCs), voltage-dependent cation channels (VDCCs), and voltage-insensitive cation channels (VICCs), have been evidenced to transport Cd^2+^ using channel blockers and flux measurements [20,21]. All these channels are characterized as nonselective cation channels (NSCCs), which are permeable to a number of cations such as Ca^2+^ and Cd^2+^ [22]. Among the above transporters, some of them, such as OsNRAMP5 and OsHMA3, have been suggested for applicability in breeding low-Cd rice via loss-of-function mutation by CRISPR (Clustered Regularly Interspaced Short Palindromic Repeats) gene editing or gene overexpression [9,15,18,19,23]. Unfortunately, very limited success has been obtained using these transporters to reduce Cd accumulation in rice under the realistic field conditions.

Apart from breeding low-Cd varieties, many other effective measures have also been taken to minimize Cd in rice. The application of exogenous substances, such as nitric oxide (NO), glutathione (GSH), selenium (Se), silicon (Si), and calcium (Ca), has been recognized as a feasible technique to alleviate Cd toxicity and reduce Cd uptake in rice [24,25,26,27,28]. Ca is a well-known essential structural, metabolic, and signaling element [22]. It plays significant roles in plant growth and development, as well as in adaptation to diverse environmental stresses [29]. It has been demonstrated that Ca plays important roles in protecting plants against Cd stress by alleviating growth inhibition, mitigating Cd-induced oxidative stress, enhancing plant photosynthesis, and changing signaling transduction [30]. Furthermore, Ca has also been reported to regulate Cd uptake and translocation in plants in a dose- and plant-species-dependent manner [30]. Gong et al. [31] found that the uptake and translocation of Cd in *Boehmeria nivea* were promoted by the treatment of 1 mM Ca, but significantly reduced by the application of 5 mM Ca. On the other hand, Eller and Brix [32] reported that a high Ca concentration (ca. 2 mM) reduced the uptake of Cd in the roots of both *Brassica juncea* and *Sesbania sesban*, but enhanced the translocation of Cd to shoots of both species, in comparison to the low Ca concentration of 0.2 mM. Moreover, in a recent study on the effect of Ca on Cd accumulation in rice with a series of Ca concentrations (0–320 mg/L), Cd concentration in rice roots was reduced by all Ca concentrations at similar level, whereas Cd concentration in rice shoots showed the highest value at 80 mg/L Ca but the lowest at 320 mg/L [33]. All these results implied that the effect of Ca on Cd uptake and translocation remains controversial and requires further investigation.

In the present study, the effect of Ca on the uptake and translocation of Cd in rice was comprehensively investigated under different growth stages, in terms of tissue Cd content, net Cd^2+^ flux, fluorescent visualization of Cd in roots, and gene expression of Cd transporters. Furthermore, the contribution of Ca channels to the uptake and translocation of Cd was analyzed through the exogenous application of channel blockers (Gd^3+^, verapamil, nifedipine, and nimodipine). With these results, we attempted to answer the following questions: (i) What is the specific role of Ca in regulating Cd uptake and translocation in rice? (ii) What is the mechanistic basis of such a specific role of Ca?

## 2. Results

### 2.1. Effect of External Ca Concentrations on Net Cd^*2+*^ Flux and Distribution of Cd in Roots and Tissue Cd Contents of Rice Plants at Seedling Stage

**Figure 1:**
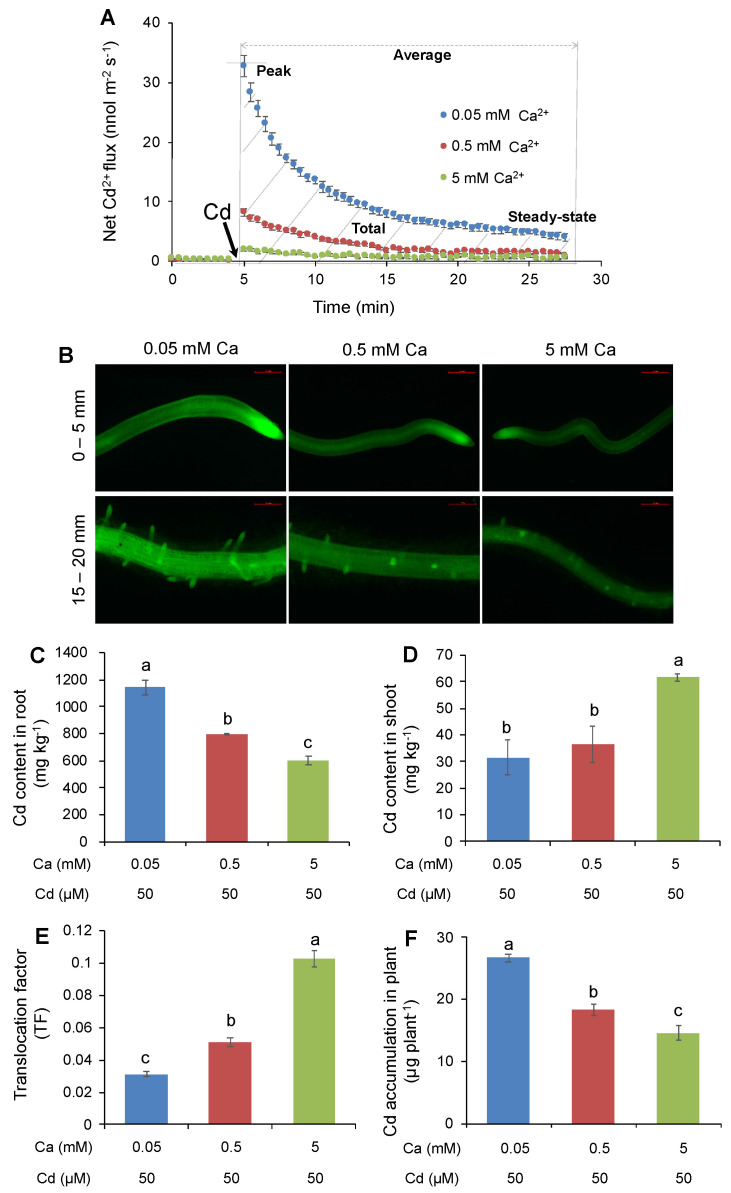
Effects of external Ca concentrations on Cd uptake, distribution, and accumulation in rice. (**A**) Transient Cd^2+^ flux in rice root epidermis under different Ca conditions; Cd treatment was applied at time as indicated by arrows. Peak flux, steady-state flux, average flux, and total flux are shown. (**B**) Visualization of Cd^2+^ in the segments of 0–5 mm and 15–20 mm from root tip under different Ca conditions by fluorescent imaging using Leadmium Green AM dye. (**C**) Cd content in rice roots. (**D**) Cd content in rice shoots. (**E**) Translocation factor (TF) of Cd from roots to shoots. (**F**) Cd accumulation in rice plant. Cd exposure: 50 μM; Ca concentrations: 0.05, 0.5, and 5 mM. Data are means ± standard error (SE) (*n* = 6–8). Different letters represent a significant difference between treatments at *p* < 0.05.

The microelectrode ion flux estimation (MIFE) technique was used to measure net Cd^2+^ flux from the rice roots (Figure 1A). Prior to adding Cd treatments, Cd^2+^ fluxes under different Ca concentrations were all kept at ~0 nmol·m^−2^·s^−1^, indicating that no Cd uptake occurred under the pretreated conditions. Addition of 50 μM Cd to the bath solution resulted in an instantaneous Cd^2+^ influx, with a peak value ranging from 2 to 33 nmol·m^−2^·s^−1^ in the mature zone under different external Ca concentrations. The influxes were then gradually reduced in all rice roots. With the external Ca concentration increasing from 0.05 mM to 5 mM, net Cd^2+^ influx was significantly decreased (*p* < 0.05), resulting in 4–40-fold lower peak, steady-state, average, and total values in the roots pretreated with 0.5 and 5 mM Ca than those in the roots pretreated with 0.05 mM Ca (Figure S1, Supplementary Materials). We next looked at the effect of Ca on Cd distribution along rice root in the segments of 0–5 mm (including meristem and elongation zones) and 15–20 mm (including mature zone) from the root cap (Figure 1B). Consistent with MIFE data, the higher external Ca concentration in the basal solution resulted in the significantly weaker intensity of Cd green fluorescence in both root segments (Figure S2, Supplementary Materials), suggesting the inhibitory effect of Ca on Cd uptake by rice roots.

The effect of Ca on Cd uptake was further investigated in terms of Cd content in rice tissues. As shown in Figure 1C, the increase in the external Ca concentration considerably reduced Cd content in rice roots, with about a 50% reduction in root Cd content under 5 mM Ca relative to that under 0.05 mM Ca. Unexpectedly, Cd contents in rice shoots were significantly enhanced with the increase in Ca concentration in the nutrient solution, with roots treated with 5 mM Ca showing twofold higher Cd content than those grown with 0.05 mM Ca (Figure 1D). As a result, a considerable increase in the translocation factor of Cd was observed when plants were exposed to higher external Ca concentration (Figure 1E). However, the accumulation of Cd in rice plant was significantly decreased with increasing Ca concentrations (Figure 1F), due to the reduction in Cd uptake by rice roots (Figure 1A–C). All these results indicated that Ca decreases root Cd uptake but facilitates root-to-shoot Cd translocation in rice.

### 2.2. Effects of Ca Channel Blockers on Net Cd^*2+*^ Flux and Distribution of Cd in Rice Roots and Tissue Cd Contents

**Figure 2:**
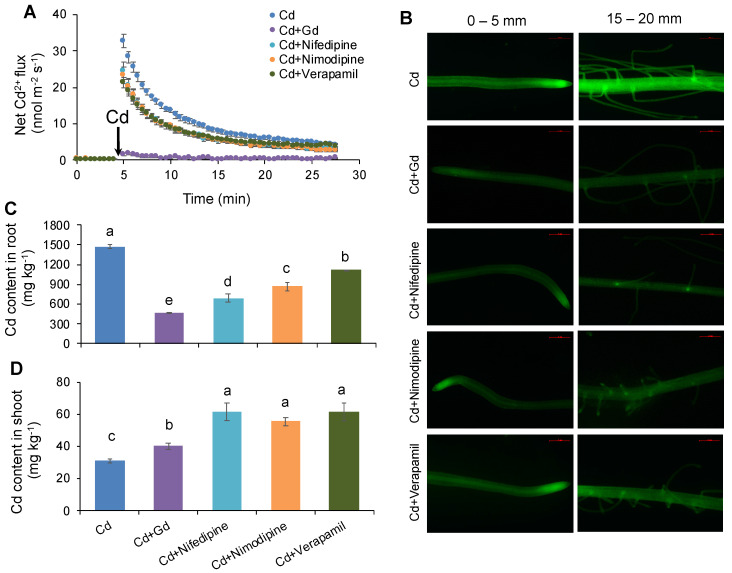
Effects of different Ca channel blockers on Cd uptake, distribution, and accumulation in rice. (**A**) Transient Cd^2+^ flux in root epidermis of rice seedlings pretreated with different Ca channel blockers. (**B**) Visualization of Cd^2+^ in the segments of 0–5 mm and 15–20 mm from root tip of rice seedlings pretreated with different Ca channel blockers by fluorescent imaging using Leadmium Green AM dye. (**C**) Cd content in rice roots. (**D**) Cd content in rice shoots. Cd exposure: 50 μM; Ca channel blockers: 100 μM Gd^3+^, nonselective cation channel (NSCC) blocker; 20 μM nifedipine, depolarization-activated calcium channel (DACC) blockers; 20 μM nimodipine, voltage-dependent cation channel (VDCC) blocker; 20 μM verapamil, hyperpolarization-activated calcium channel (HACC) blocker. Data are means ± SE (*n* = 6–8). Different letters represent a significant difference between treatments at *p* < 0.05.

The effects of Ca channel blockers on net Cd^2+^ flux kinetics were investigated to reveal the possibility of Ca channels mediating root Cd uptake. All the blockers used in this study induced little changes in the initial Cd^2+^ flux after 1 h of incubation, whereases they showed significantly inhibitory effects on the net Cd^2+^ influx (Figure 2A). To show the difference between treatments, the reduction relative to the treatment of Cd alone was calculated for each blocker according to the results presenting in the figures. Gd^3+^ is a well-known blocker of NSCCs [34]. As expected, pretreatment of 100 μM Gd^3+^ resulted in a drastic reduction in net Cd^2+^ influx. The peak, steady-state, average, and total Cd^2+^ influxes of Gd^3+^-pretreated rice roots were reduced by 95% relative to those treated with Cd alone (Figure S3, Supplementary Materials). To specify which type of Ca channel mediates Cd uptake in rice roots, the net Cd^2+^ flux kinetics were further measured in the presence of three different Ca channel blockers: nifedipine (DACC blocker) [35], nimodipine (VDCC blocker) [36], and verapamil (HACC blocker) [37]. As shown in Figure 2A, all three blockers significantly reduced the net Cd^2+^ influx in rice roots, whereas they showed much lower inhibitory effects than Gd^3+^ did. The peak, steady-state, average, and total Cd^2+^ influxes of rice roots were only reduced by 18.4–27.3%, 29.0–40.6%, and 11.9–35.0% in the presence of nifedipine, nimodipine, and verapamil respectively, relative to the treatment of Cd alone (Figure S3, Supplementary Materials). Moreover, the inhibitory effects of these three blockers on net Cd^2+^ influxes were quite similar, indicating that DACCs, VDCCs, and HACCs might have a similar contribution to net Cd^2+^ influx in rice, e.g., about 30% on average for the three blockers in the present study (Figure 2A and Figure S3, Supplementary Materials).

The effects of Ca channel blockers on Cd uptake were further verified by fluorescent labeling of Cd ions along rice roots after 24 h of Cd exposure. As expected, the presence of pharmaceuticals significantly reduced the Cd green fluorescence in both root segments of 0–5 mm and 15–20 mm from the root cap (Figure 2B). The inhibitory effects of each pharmaceutical were quite similar in both root segments (Figure S4, Supplementary Materials). By calculating the reduction in the average density of Cd fluorescence relative to the treatment of Cd alone, the inhibitory effects of four pharmaceuticals were shown in the order of Gd^3+^ (70.4%) > nimodipine (57.4%) > nifedipine (38.5%) ~ verapamil (31.6%) on average in two root segments (Figure S4, Supplementary Materials).

Total Cd contents were determined in the bulk roots of rice seedlings pretreated with different pharmaceuticals after 3 days of Cd exposure to verify the results of MIFE measurements and Cd fluorescence detection. Pretreatment with Gd^3+^, nimodipine, nifedipine, and verapamil significantly restricted the accumulation of Cd in rice roots (Figure 2C). Likewise, Gd^3+^ showed the strongest inhibition in root Cd content by 71.2% compared to the treatment of Cd alone (Figure 2C), in agreement with the results Cd fluorescent detection (70.4%) but less than the MIFE measurements (95%; Figure S3, Supplementary Materials). However, the inhibitory effects of nimodipine, nifedipine, and verapamil were much smaller, with an order of nifedipine (52.9%) > nimodipine (40.9%) > verapamil (24.0%), as compared with the treatment of Ca alone (Figure 2C).

In addition, it was noted that Ca channel blockers did not reduce but significantly increased shoot Cd contents (Figure 2D), being consistent with the effect of increasing external Ca concentration (Figure 1D). Of all four blockers, Gd^3+^ showed the smallest increase in shoot Cd content, and no significant difference was observed among the other three blockers (Figure 2D). These results indicated that the translocation of Cd from root to shoot was highly enhanced by the presence of Ca channel blockers, but in a blocker-specific manner.

### 2.3. Gene Expression under Different Ca Concentrations or Pharmacological Treatments

**Figure 3:**
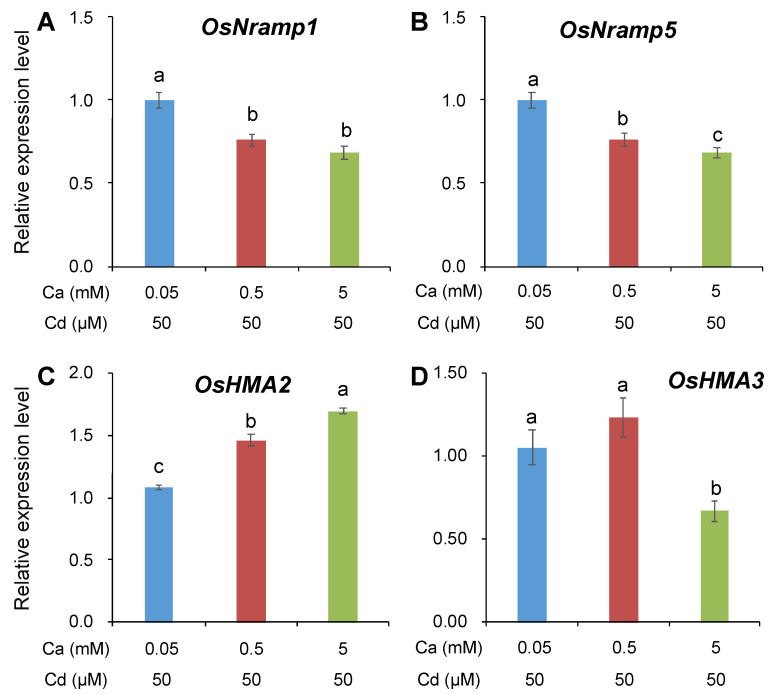
Gene expression of *OsNRAMP1* (**A**), *OsNRAMP5* (**B**), *OsHMA2* (**C**), and *OsHMA3* (**D**) in rice roots supplied with different Ca concentrations in response to Cd exposure. Cd exposure: 50 μM; Ca concentrations: 0.05, 0.5, and 5 mM. Gene expression in roots under 0.05 mM Ca concentration was normalized to 1, and gene expressions with the other Ca concentration were compared relative to it. Data are means ± SE (*n* = 3 biological replicates). Different letters represent a significant difference between treatments at *p* < 0.05.

**Figure 4:**
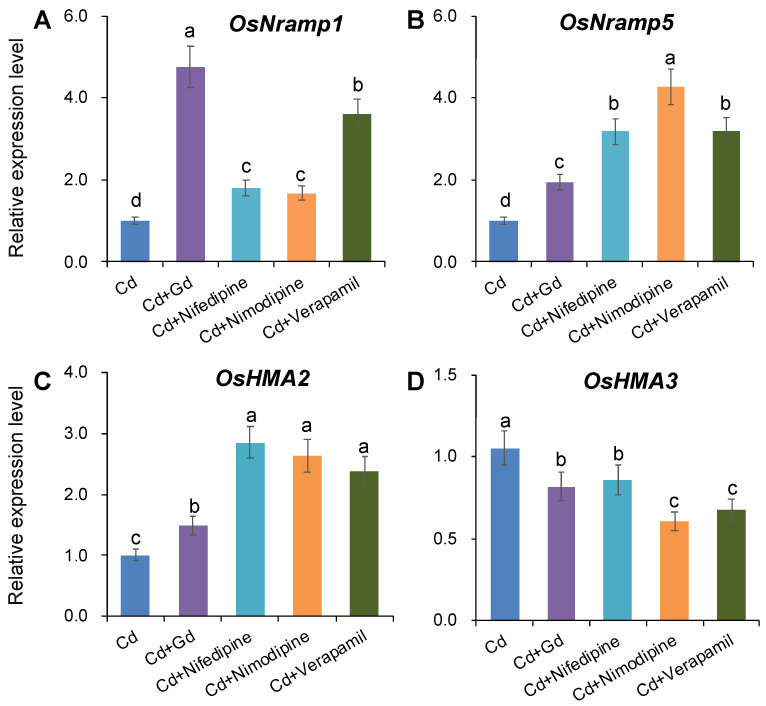
Gene expression of *OsNRAMP1* (**A**), *OsNRAMP5* (**B**), *OsHMA2* (**C**), and *OsHMA3* (**D**) in rice roots pretreated with different Ca channel blockers in response to Cd exposure. Cd exposure: 50 μM; Ca channel blockers: 100 μM Gd^3+^, NSCC blocker; 20 μM nifedipine, DACC blockers; 20 μM nimodipine, VDCC blocker; 20 μM verapamil, HACC blocker. Gene expression in root treated with Cd alone was normalized to 1, and gene expressions with different Ca channel blockers were compared relative to it. Data are means ± SE (*n* = 3 biological replicates). Different letters represent a significant difference between treatments at *p* < 0.05.

The gene expression of four major plasma transporters controlling Cd uptake in rice roots (OsNRAMP1 and OsNRAMP5) and Cd translocation from root to shoot (OsHMA2 and OsHMA3) was determined by qRT-PCR after onset of 50 µM Cd for 3 days, under different Ca concentrations or pharmacological treatments (Figure 3 and Figure 4). With the increase in external Ca concentration, the expression of both *OsNRAMP1* and *OsNRAMP5* was significantly reduced by 23.0–33.0% (Figure 3A,B). Surprisingly, however, the expression of these two NRAMP genes was not inhibited but induced by all pharmacological treatments (Figure 4A,B). These results indicated that the Ca-mediated reduction in root Cd uptake might be attributed to the suppression of gene expression of *OsNRAMP1* and *OsNRAMP5*, whereas the pharmacological treatment-induced inhibition in Cd uptake had little association with the expression of these genes.

On the other hand, the case was quite different for *OsHMA2* and *OsHMA3*. The increasing Ca concentration in the external solution significantly induced the expression of *OsHMA2* in rice roots (Figure 3C), suggesting an enhancement in xylem loading of Cd in rice roots by Ca. Furthermore, the expression of *OsHMA3* was suppressed by 5 mM Ca (Figure 3D), indicating a decrease in Cd sequestration into vacuoles under the condition of high Ca. Likewise, the different pharmacological treatments were observed to induce the expression of *OsHMA2* but reduce the expression of *OsHMA3* (Figure 4C,D). All these suggested that Ca ions or Ca channel blockers could not only reduce the sequestration of Cd into vacuoles but also induce the loading of Cd into xylem, thereby facilitating Cd translocation from root to shoot.

### 2.4. Effects of External Ca Concentration on Plant Growth and Tissue Cd Content in Rice Plants at Tillering and Ripening Stages

To further verify the effect of Ca on the growth and the uptake and translocation of Cd in rice plants during the whole growth period, we next determined the plant height, biomass, and Cd contents in rice tissues at tillering and ripening stages. As shown in Figures S5 and S6 (Supplementary Materials), the growth of rice plants was significantly inhibited by the exposure of Cd. The increase in external Ca concentration from 0.5 to 5 mM greatly alleviated the adverse impact of Cd on rice plant height elongation and tissue biomass accumulation at both tillering and ripening stages. On the contrary, the decrease in external Ca concentration from 0.5 to 0.05 mM dramatically deteriorated the adverse impacts of Cd on rice plant growth, which resulted in the death of rice plants after the onset of Cd exposure for only one month, such that no data were obtained for the treatment of 0.05 mM Ca + 50 μM Cd at the ripening stage (Figures S5 and S6, Supplementary Materials).

**Figure 5:**
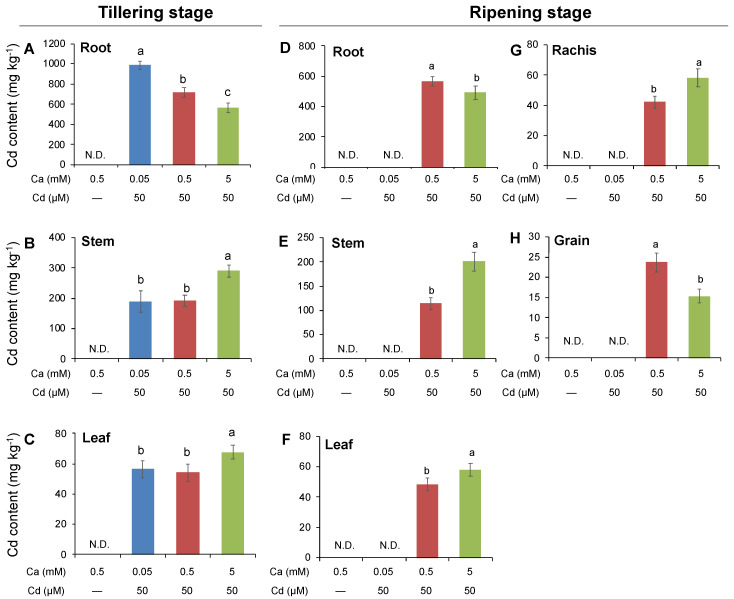
Effects of external Ca concentrations on tissue Cd content in rice plants at the tillering and ripening stage. (**A**–**C**) Cd contents in root, stem, and leaf of rice plants at the tillering stage; (**D**–**H**) Cd contents in the root, stem, leaf, rachis, and grain of rice plants at the ripening stage. Cd exposure: 50 μM; Ca concentrations: 0.05, 0.5, and 5 mM. Data are means ± SE (*n* = 6). Rice plants grown with 0.05 mM Ca + 50 μM Cd died after 1 month of the treatment, such that data at ripening stage were not detected (N.D.). Different letters represent a significant difference between treatments at *p* < 0.05.

Being consistent with the results at the seedling stage, the increase in external Ca concentration from 0.5 to 5 mM significantly decreased the Cd content in roots (Figure 5A,D) but increased the Cd content in stems (Figure 5B,E) and leaves (Figure 5C,F) of rice plants at both tillering and ripening stages. However, the decrease in external Ca concentration from 0.5 to 0.05 mM dramatically increased the Cd content in root but caused little change in the Cd content in stems and leaves at the tillering stage (Figure 5B,C). No result for Cd content in rice tissues under the treatment of 0.05 mM Ca + 50 μM Cd was available at the ripening stage because the plants died 1 month after Cd exposure (Figure 5D–H). Furthermore, rice panicles were harvested at the ripening stage and separated into rachises and grains to determine their Cd content. Notably, in comparison to 0.05 mM Ca, 5 mM Ca significantly increased the Cd content in rachises (Figure 5G, *p* < 0.05), but greatly decreased the Cd content in grains by 35.4% (Figure 5H, *p* < 0.05). These results indicated that Ca could significantly affect the allocation of Cd from the vegetative parts to reproductive ones of rice plants.

## 3. Discussion

### 3.1. Ca Plays a Double-Edged Role in Regulating Root Cd Uptake and Root-To-Shoot Cd Translocation in Rice

Calcium is an essential element crucial for plant growth and development under both non-stressed and stress conditions [29]. It is well documented that Ca plays a significant role in alleviating Cd toxicity in plants [30]. Inhibition of growth and development is one of the distinct symptoms of Cd toxicity. It has been extensively reported that the exogenous application of Ca could positively affect plant height, root length, and biomass production of plants under Cd stress [30,38]. In the present study, an elevating Ca concentration (ca. from 0.5 mM to 5 mM) in the nutrient solution was also observed to significantly ameliorate the Cd-induced reduction in plant height and biomass in rice plants at both tillering and ripening stages (Figures S5 and S6, Supplementary Materials).

Possible mechanisms suggested for the protective role of Ca against Cd-induced toxicity in plants include mitigating Cd-induced oxidative stress, enhancing plant photosynthesis, changing signaling transduction, and especially decreasing Cd uptake and accumulation [39,40]. However, many studies found that the effects of Ca on Cd uptake, translocation, and accumulation in plants are complex and dependent on Ca doses and plant species [30,36]. Gong et al. [31] reported that the uptake and translocation of Cd in *Boehmeria nivea* were promoted by 1 mM Ca, but reduced by 5 mM Ca. On the other hand, in *Brassica juncea* and *Sesbania sesban*, root Cd uptake was reduced by the increasing Ca concentration from 0.2 mM to 2 mM, but root-to-shoot Cd translocation was enhanced [32]. Recently, Ye et al. [33] reported that the application of 80 mg/L (~2 mM) Ca increased Cd content in the shoot but caused little change in Cd content in the root of rice plants, resulting in a significant increase in translocation factor. In the present study, the effects of Ca on Cd uptake and translocation were investigated by measuring net Cd^2+^ fluxes from the epidermis of root mature zone, labeling Cd ions along roots, and determining Cd content in rice tissues at the seedling, tillering, or ripening stage. Our results showed that, at the seedling stage, the increase in external Ca concentration from 0.05 to 5 mM significantly reduced net Cd^2+^ influx by the root epidermis (Figure 1A), Cd green fluorescence in root segments (Figure 1B), and the Cd content in the bulk roots of rice plants (Figure 1C), but dramatically enhanced the Cd content in rice shoots and the translocation factor of Cd (Figure 1D,E). With rice plants growing to the tillering and ripening stages, similar results were observed for Cd contents in the roots and aboveground vegetative tissues (Figure 5). Taken together, all these results revealed that Ca decreases root Cd uptake but facilitates root-to-shoot Cd translocation, suggesting a double-edged role of Ca in regulating Cd uptake and translocation in rice.

### 3.2. Ca Decreases Root Cd Uptake in Rice Not Only by Competing for Absorption Sites but Also by Suppressing the Expression of OsNRAMPs

Although rice plants exhibit a relative high capacity of Cd absorption and accumulation, the nonessentiality and toxicity of Cd to plants make its uptake by rice roots a deep mystery [41]. To our best knowledge, there is no confirmed specific transporter for Cd uptake in plants so far. It is well known that Cd^2+^ shares many chemical similarities with Ca^2+^, such that competition probably occurs between these two ions in their uptake by plants [20,21]. Indeed, increasing evidence has revealed that Cd could compete with Ca for Ca channels or NSCC channels to enter the plasma membrane of root cells in various plant species [21,42,43,44,45]. As expected, net Cd^2+^ influx, Cd green fluorescence, and the Cd content in rice roots were all drastically reduced by the NSCC blocker Gd^3+^, with the reductions ranging from 70% to 95% (Figure 2 and Figures S3 and S4, Supplementary Materials), once again implying the involvement of NSCC channels in Cd uptake into rice roots. Various NSCC channels permeable to Ca have been postulated to mediate Cd transport in plant roots, including DACCs, HACCs, VDCCs, and VICCs, which differ in their sensitivity to membrane voltage [21,44,46]. Of them, VICC channels such as glutamate-like receptors (OsGLRs) have been evidenced to play an important role in Cd uptake into rice roots [21]. In the present study, the contribution of the other three kinds of channels to Cd uptake in rice roots was specified by applying different Ca-permeable channel blockers. Our results revealed that nifedipine (DACC blocker) [35], nimodipine (VDCC blocker) [36], and verapamil (HACC blocker) [37] significantly inhibited Cd uptake into rice roots with a reduction of about 40% on average, and their inhibitory effects were relatively similar (Figure 2). All these results suggested that Cd could compete with Ca for DACCs, VDCCs, and HACCs to enter root cells in rice, but the contribution of these channels to Cd uptake is limited at around 40%. Therefore, the contribution of all above channels to Cd uptake into rice root should not be ignored, and further studies are needed to identify the novel channels controlling Cd uptake in rice.

As mentioned above, the inhibitory effect of Ca on Cd uptake is partially attributed to their competition for ion channels as uptake routes. In this case, therefore, there are definitely other possible mechanisms conferring the Ca-induced inhibition in Cd uptake. To address this issue, the gene expression of *OsNRAMP1* and *OsNRAMP5*, which are considered to be crucial contributors to Cd entering into rice roots [9,15], was determined under different Ca concentrations or pharmacological treatments in the present study. Increasing the external Ca concentration significantly downregulated the expression of *OsNRAMP1* and *OsNRAMP5* (Figure 3A,B). Likewise, Treesubsuntorn and Thiravetyan [47] also reported a downregulation of *OsNRAMP5* in rice plants upon applying calcium acetate (Ca(CH_3_COO)_2_) in soil. These results proved that Ca could also decrease Cd uptake into rice roots by suppressing the expression of *OsNRAMP1* and *OsNRAMP5*. Moreover, an interesting result was observed that the expression of *OsNRAMP1* and *OsNRAMP5* was upregulated by the all pharmacological treatments (Figure 4A,B), indicating that the inhibitory effect of Ca channel blockers on Cd uptake in rice roots was not directly related to the expression of *OsNRAMP1* and *OsNRAMP5*. Similar results were also observed in previous studies [21,22]. There are two possible reasons for the upregulation of *OsNRAMP1* and *OsNRAMP5* by Ca channel blockers. First, all pharmaceuticals in this study could trigger membrane potential depolarization, which activates plasma membrane H^+^-ATPase, thereby stimulating the expression of *OsNRAMPs* [22]. Second, the upregulation of *OsNRAMPs* may result from the pharmaceutical-reduced Ca-mediated H_2_O_2_ generation, which could downregulate Cd transporter genes [48]. However, the related mechanistic basis remains to be answered in future studies.

### 3.3. Ca Facilitates Root-To-Shoot Cd Translocation in Rice by Modulating the Expression of OsHMAs

Apart from Cd uptake in the roots, the translocation of Cd from roots to shoots is another critical process controlling the accumulation of Cd in the aboveground tissues of rice plants [1,49]. It has been reported that Ca treatment reduced Cd accumulation in the aboveground parts of rice and *Gamblea innovans* [47,50]. On the contrary, many other studies revealed that Ca could also induce Cd translocation from roots to shoots in *Arabidopsis* [51], chamomile [52], and rice [33]. Such contrary results make it important to further specify the effect of Ca on Cd translocation from roots to shoots. In this study, the effect of Ca on Cd translocation from roots to shoots in rice plants was investigated by determining Cd content in different tissues at the seedling, tillering, and ripening stages. It was quite consistently observed that increasing the external Ca concentration (especially from 0.5 to 5 mM) greatly increased the Cd content in all the aboveground vegetative tissues at all three growth stages (Figure 1D and Figure 5B–G), suggesting the facilitation of Ca in Cd translocation from roots to shoots in rice plants. To explain its mechanistic basis, we further determined the gene expression of *OsHMA2* and *OsHMA3*, which are the major contributors to xylem loading of Cd [19,53] and Cd compartmentation into vacuoles in root cells [18,54], respectively. Our results showed that increasing the Ca concentration from 0.5 to 5 mM significantly induced the expression of *OsHMA2* in rice roots (Figure 3C), suggesting the promotion of Ca in loading Cd into the xylem, whereas it suppressed the expression of *OsHMA3* (Figure 3D), indicating a decrease in Cd sequestration into vacuoles. Similar results were obtained in the previous studies on *Arabidopsis* [51] and rice [33]. In addition, Ca channel blockers were observed to upregulate the expression of *OsHMA2* but downregulate the expression of *OsHMA3* (Figure 4C,D). Taken together, it can be concluded that Ca or Ca channel blockers can not only induce the loading of Cd into xylem but also reduce the sequestration of Cd into vacuoles, thereby facilitating the upward translocation of Cd from roots to shoots in rice.

### 3.4. The Potentiality of Ca-Based Amendments for Low-Cd Rice Production

Soil contamination with Cd poses a great threat to animals and plants; thus, the remediation of Cd-contaminated soils to produce low-Cd crops, thereby preventing their entry into the food chain, is of high concern. Different chemical, physical, and biological approaches, including soil amendments [55], fertilizer management [56], water management [57], tillage management [58], phytoremediation with hyperaccumulator plant species [59], microbial remediation [3], and molecular modification of crops via loss-of-function mutation by CRISPR gene editing or gene overexpression [9,15,18,19,23], have been extensively employed to immobilize or reduce Cd in soil and eventually mitigate Cd uptake into rice. As compared to other strategies, inorganic amendments are effective, cost-saving, and environmentally friendly to lower Cd bioavailability in Cd-contaminated soils because of their environmentally compatible characteristics [60]. Among inorganic amendments, Ca compounds, such as calcite (CaCO_3_), burnt lime (CaO), dolomite CaMg(CO_3_)_2_, slag (CaSiO_3_), and slaked lime Ca(OH)_2_, have been widely used in paddy soils to reduce Cd uptake by rice [33,61,62]. The major factor giving rise to their role in reducing Cd uptake has been explained as the above Ca amendments potentially increasing soil pH and the stabilization of Cd in contaminated soils [60]. On the other hand, increasing evidence has also shown that the application of Ca regulates Cd uptake, translocation, and accumulation in plants [30,31,32,33,47,48,49,50,51,52]. Indeed, the present study revealed that the high Ca concentration in the nutrient solution could dramatically reduce the net Cd^2+^ influx and Cd content in rice roots (Figure 1 and Figure 5A,D), further proving the importance of Ca in reducing Cd uptake in rice. On the other hand, however, Ca addition could also promote the upward translocation of Cd from roots to shoots, as shown by much higher Cd contents in the aboveground vegetative tissues under high Ca (Figure 1D and Figure 5B,C,E–G). These results may have relevance for the bioremediation of Cd-contaminated soils with the integration of hyperaccumulators and Ca amendments [31,51]. However, this hypothesis should be further verified with a number of hyperaccumulators by investigating the effect of Ca amendments on their Cd uptake and translocation in both bench-scale and field experiments. Furthermore, it is worth mentioning that, although Ca facilitated the upward translocation of Cd, its application eventually decreased the Cd accumulation in rice grains at the ripening stage (Figure 5H), implying the possibility of producing low-Cd rice by Ca application. Taken together, it can be concluded that the application of Ca-based amendments exhibits great potential for the production of low-Cd rice.

## 4. Materials and Methods

### 4.1. Plant Materials, Growth Conditions, and Treatments

Rice cultivar Xiushui 09 used in this study was obtained from China National Rice Research Institute (Hangzhou, China). Seeds were surface-sterilized with 2% H_2_O_2_ for 15 min and thoroughly rinsed with tap water for 30 min. The sterilized rice seeds were then soaked in distilled water at 25 °C for 2 days and subsequently germinated with limited water at 35 °C for another day. The well-germinated seeds were sown on floating mesh in a container with Yoshida rice nutrient solution according to Zeng et al. [63] with some modifications. The concentration of Ca in the solution was set to 0.5 mM, and the pH value of nutrient solution was adjusted to 5.60 using 1 M HNO_3_ or KOH solution as required. Rice seedlings were grown in a well-controlled growth chamber with a photoperiod of 16 h light/8 h dark, a temperature of 32 °C light/28 °C dark, a light intensity of 225 ± 25 µmol·m^−2^·s^−1^, and a relative humidity of 80%.

For the experiment at the seedling stage, rice seedlings were grown in the nutrient solution. After 15 days, the uniform and healthy seedlings were transplanted to the basal salt medium solution (BSM, 1 mM KNO_3_ + 0.5 mM Ca(NO_3_)_2_) for 1 day to get rid of the ionic interferences in the electrophysiological measurements. Rice seedlings were then used for electrophysiological measurements or were exposed to the following Ca and Cd treatments in the background of BSM for further investigations: 0.05 mM Ca + 50 μM Cd, 0.5 mM Ca + 50 μM Cd, and 5 mM Ca + 50 μM Cd. Both Ca and Cd stock solutions were prepared with Ca(NO_3_)_2_ and Cd(NO_3_)_2_ in the background of BSM, respectively. Solutions were changed every day. After onset of treatments for 3 days, rice seedlings were collected and separated into roots and shoots for the measurements of Cd contents and gene expression analysis.

For the experiments of the whole growth period, rice seedlings were grown in a 10 L container with nutrient solution as described above to the five-leaf stage. Then, they were exposed to four Ca and Cd treatments: 0.5 mM Ca (same as the concentration in the Yoshida rice nutrient solution, used as the control), 0.05 mM Ca + 50 μM Cd, 0.5 mM Ca + 50 μM Cd, and 5 mM Ca + 50 μM Cd. Solutions were changed every 3 days. When they were grown to the tillering stage and ripening stage, rice plants were recorded for their height and biomass and then separated into roots, stems, leaves, rachises, and grains to measure the total Cd content.

### 4.2. Measurement of Cd Content in Rice Tissues

The collected rice tissues were dried at 70 °C to constant weight for recording their dry biomass. The dried tissues were incubated overnight with concentrated nitric acid and perchloric acid (3:1 *v*/*v*) and subsequently digested for 1 h at 150 °C and another 1 h at 180 °C using a Dry Thermo Unit (Taitec, Tokyo, Japan). The digested solutions were diluted with deionized water and determined for Cd concentration by using an atomic absorption spectrophotometer (AA6300, Shimadzu, Tokyo, Japan), with 1000 mg·L^−1^ Cd in 2% nitric acid (cat. 51994, Sigma-Aldrich, Shanghai, China) as a certified reference material. Tissue Cd contents were then calculated with the Cd concentrations in the diluted solution and tissue dry weights. The translocation factor (TF) was calculated as follows:

TF = Cd content in shoot/Cd content in root.(1)

The Cd accumulation in plant was calculated as follows:

Cd accumulation in plant (µg·plant^−1^) = ∑ tissue Cd content (mg·kg^−1^) × tissue dry weight (g·plant^−1^). (2)

### 4.3. Microelectrode Measurements of Net Cd^*2+*^ Flux

#### 4.3.1. Procedure of Microelectrode Ion Flux Measurements

Net Cd^2+^ fluxes were measured using noninvasive ion-selective vibrating microelectrodes (the MIFE technique, University of Tasmania, Hobart, Australia). The basic principle and measuring procedure of MIFE technique were extensively described in detail elsewhere [64]. Briefly, the microelectrodes were tip-excised to 2 to 3 µm in diameter, and then back-filled with an electrolyte buffer (0.1 mM KCl + 10 mM Cd(NO_3_)_2_) and front-filled with an ion-selective Cd^2+^ cocktail according to Piñeros et al. [65]. Prior to the measurement, the prepared Cd^2+^ microelectrodes were stabilized for at least 2 h and calibrated in a set of the standard solutions: 25, 50, and 100 μM Cd, prepared with Cd(NO_3_)_2_. Only electrodes with Nernstian slopes >25 mV and correlation ≥0.9990 were used. Ion fluxes were measured by a slow (6 s half-cycle) square-wave movement of microelectrodes between two positions, close to (50 µm) and away from (100 µm) the root surface. The voltage difference between two positions was recorded by the MIFE CHART software and converted to the electrochemical potential difference using the calibrated Nernst slope of the electrodes. Net ion fluxes were then calculated from the electrochemical potential difference using cylindrical diffusion geometry with the MIFEFLUX program [64,66] using the following formulas:

net Cd^2+^ flux (*J*) = c × u × (d*µ*/d*x*), (3)

where c is the ion concentration (mol·m^−3^), and u is the ion mobility (m·s^−1^ per N·mol^−1^);

d*µ* = z × F × *g* × d*V*, (4)

where z is the ion’s valence, F is the Faraday number (96,500 °C·mol^−1^), *g* is the Nernst slope for the electrode during calibration, and d*V* is the voltage difference between the two positions (V);

d*x* = r × ln [(r + x + dx)/(r + x)], (5)

where r is the radius of cylinder (m), x is the distance of position 1 from the root surface (m), and dx is the distance of position 2 from position 1 (m).

#### 4.3.2. Transient Cd^2+^ Flux Kinetics

Roots (≥8 cm) of BSM-stabilized intact rice seedlings were mounted in a horizontal chamber 1 h prior to the measurements and pretreated with one of the following solutions: 1 mM KNO_3_ + 0.05 mM Ca(NO_3_)_2_, 1 mM KNO_3_ + 0.5 mM Ca(NO_3_)_2_, or 1 mM KNO_3_ + 0.5 mM Ca(NO_3_)_2_. Net Cd^2+^ flux was measured for 5 min under each pretreated condition to record the steady initial flux values. Subsequently, the measuring solution was replaced with the 50 μM Cd(NO_3_)_2_ in the corresponding background of the above pretreated solution, and the transient ion flux responses were measured for another 25 min. The period of time for replacing the solution (~1 min) was omitted from the data analysis and figures. Net ion fluxes were measured from the mature root (~20 mm from the root cap, with root hair) epidermis. Eight individual seedlings were measured for each treatment.

#### 4.3.3. Pharmacological Experiments

To reveal the possibility of Ca channels mediating root Cd uptake, the effects of Ca channel blockers on net Cd^2+^ flux kinetics were investigated in this study. In pharmacological experiments, roots of the BSM-stabilized intact rice seedlings were pretreated for 1 h prior to the measurements with one of the following pharmacological solutions: 100 μM GdCl_3_ (Gd, nonselective cation channel blocker, NSCC blocker) [34], 20 μM nifedipine (depolarization-activated Ca^2+^ channel blocker, DACC blocker) [35], 20 μM nimodipine (voltage-dependent Ca^2+^ channel blocker, VDCC blocker) [36], and 20 μM verapamil (hyperpolarization-activated Ca^2+^ channel blocker, HACC blocker) [37]. Net Cd^2+^ fluxes were first measured for 5 min under the pretreated pharmacological conditions. Subsequently, the measuring solution was replaced with the 50 μM Cd(NO_3_)_2_ in the corresponding background of the above pharmacological solution, and the transient ion flux responses were measured for another 30 min. Six individual seedlings were measured for each treatment. The measured rice seedlings were then regrown under the corresponding pharmacological conditions for another 3 days to determine Cd contents in rice tissues as described above.

### 4.4. Histochemical Detection of Cd in Rice Root

BSM-stabilized intact rice seedlings were pretreated with different Ca concentrations or pharmaceuticals for 1 h, and then given an appropriate volume of Cd(NO_3_)_2_ stock solution to yield a final Cd concentration of 50 μM. Rice plants were subsequently treated for 24 h prior to the detection. The in situ localization of Cd in rice roots was detected using Cd Probe Leadmium Green AM dye (Molecular Probes, Invitrogen, Calsbad, CA, USA) according to Chen et al. [21]. Cd green fluorescence were observed and analysis from root segments of 0–5 mm and 15–20 mm from the root cap.

### 4.5. RNA Extraction and Gene Expression Analysis

The transcript levels of genes controlling Cd uptake (*OsNRAMP1*, *OsNRAMP5*) and translocation (*OsHMA2* and *OsHMA3*) were determined in the bulk rice roots treated with 50 μM Cd in the presence of different Ca concentrations and pharmaceuticals. After onset of the treatments for 3 days, the bulk roots of each treatment were collected and immediately frozen in liquid nitrogen for total RNA extraction. Total RNA was isolated by the MiniBEST Plant RNA Extraction Kit (TaKaRa, Kyoto, Japan). Quantitative real-time PCR (qRT-PCR) was performed using a Light Cycler 480 Ⅱ (Roche Molecular Systems, Inc., Basel, Switzerland) with the iTaq^TM^ Universal SYBR Green Supermix (Bio-Rad Laboratories, Inc., Hercules, CA, USA). Sequences of primers for qRT-PCR are given in Table S1 (Supplementary Materials). Three biological replicates were measured for each treatment, with three technical replicates conducted for each biological replicate. The relative gene expression was calculated using the 2^−ΔΔCt^ method with *OsActin* as the internal standard [67].

### 4.6. Statistics Analysis

Statistical analysis was performed using SPSS Statistics 20 (IBM, New York, NY, USA). All data in the figures were given as means ± SE. The significant difference between means was evaluated by ANOVA. Significant differences among the means were compared using Tukey’s multiple range tests at *p* < 0.05.

## 5. Conclusions

In conclusion, external addition of Ca significantly reduced Cd uptake into rice roots, mainly by competing for Ca-permeable channels as an absorption site, as well as suppressing the expression of *OsNRAMP1* and *OsNRAMP5*. However, Ca addition facilitated the upward translocation of Cd from roots to shoots by upregulating the expression of *OsHMA2* to induce the loading of Cd into xylem and downregulating the expression of *OsHMA3* to reduce the sequestration of Cd into vacuoles. These results suggested a double-edged role of Ca in regulating Cd uptake and translocation in rice. Furthermore, although Ca increased the accumulation of Cd in the aboveground vegetative tissues during the whole growth period, its addition eventually decreased the Cd accumulation in rice grains at the ripening stage, implying the great potential of Ca-based amendments in the production of low-Cd rice to reduce Cd intake in populations. However, further studies are needed to obtain more mechanistic insights into the interactions between Ca and Cd using a broad range of plant species. Moreover, the optimal conditions, e.g., Ca concentration, soil properties, and climate, need to be determined so as to promote the remediation of Cd-contaminated soils and produce low-Cd crop products.

## Supplementary Materials

Supplementary materials can be found at https://www.mdpi.com/1422-0067/21/21/8058/s1. Figure S1. Effects of external Ca concentrations on the characteristics of net Cd^2+^ flux from rice root epidermis; Figure S2. Average density of Cd green fluorescence in the root segments under different Ca concentrations; Figure S3. Effects of different Ca channel blockers on the characteristics of net Cd^2+^ flux from rice root epidermis; Figure S4. Average density of Cd green fluorescence in the root segments under different Ca concentrations; Figure S5. Effect of external Ca concentrations (0.05 mM, 0.5 mM and 5 mM) on the growth of rice plants with or without Cd stress; Figure S6. Effect of external Ca concentrations (0.05 mM, 0.5 mM and 5 mM) on biomass accumulation of rice plants with or without Cd stress; Table S1. Primer sequences used for qRT-PCR.
