# Supplementary material for: Calcium Plays a Double-Edged Role in Modulating Cadmium Uptake and Translocation in Rice
Source: Int J Mol Sci. 2020 Oct 29;21(21):8058. doi: 10.3390/ijms21218058 (PMC7662230; doi:10.3390/ijms21218058)
Supplement: Supplementary file 1 [file ijms-21-08058-s001.zip › Supplementary Figures Final.pdf]

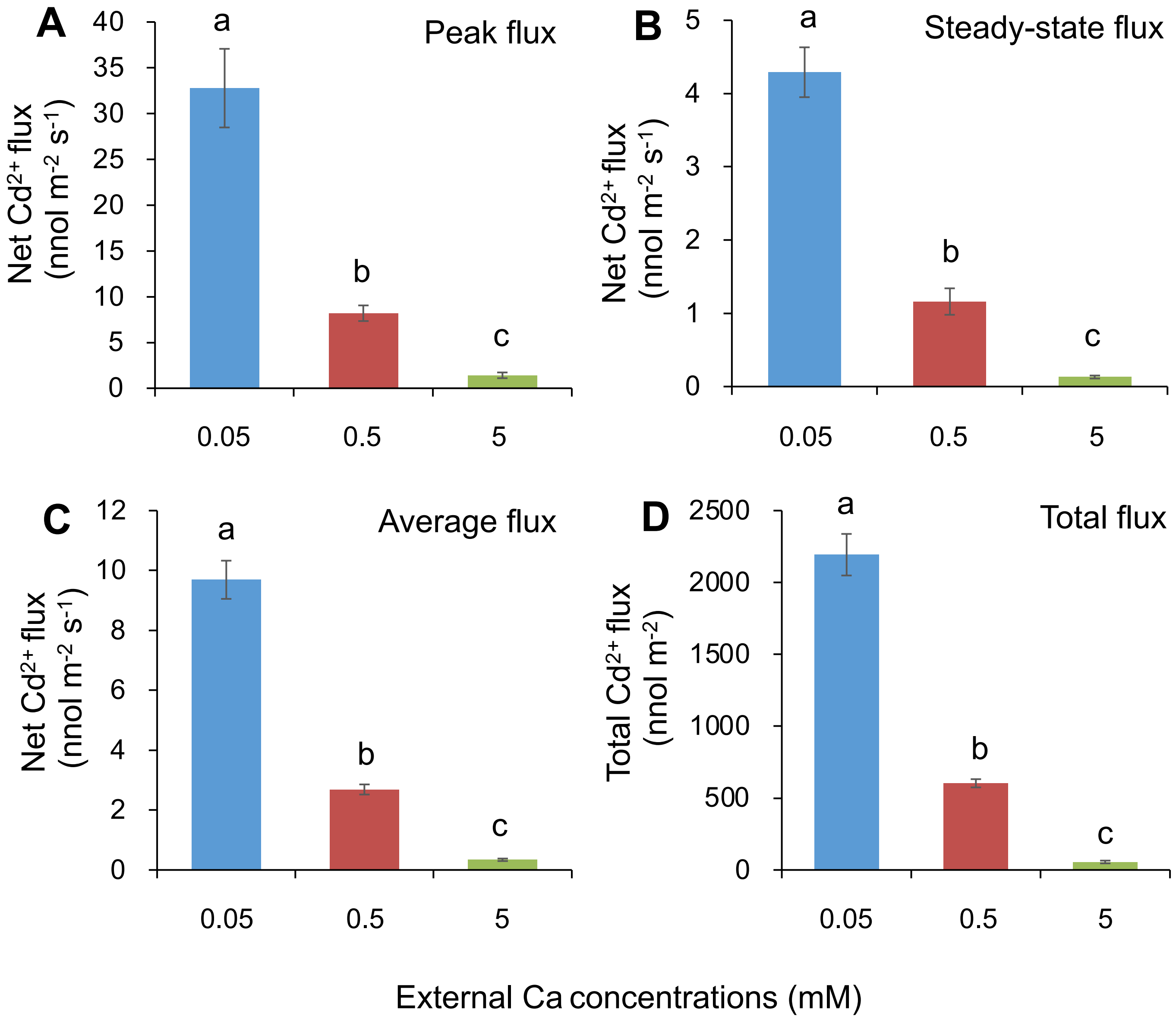

Figure S1. Effects of external Ca concentrations on the characteristics of net Cd^{2+} flux from rice root epidermis. (**A**), Peak Cd^{2+} flux immediately after Cd treatment; (**B**), Steady-state Cd^{2+} flux at the end of the flux measurement; (**C**), Average Cd^{2+} flux over 25 min after Cd treatment; and (**D**), Total Cd^{2+} flux summed over 25 min after Cd treatment. Cd exposure: 50 μM; Ca concentrations: 0.05, 0.5 and 5 mM. Data are means ±SE (n=8). Different letters represent the significant difference between treatments at $P<0.05$.

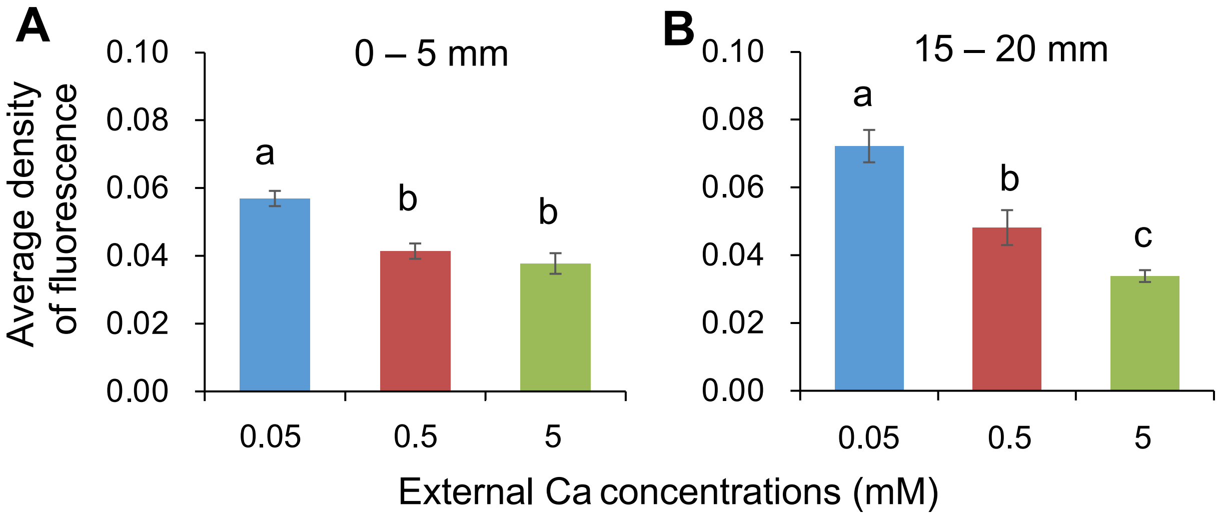

Figure S2. Average density of Cd green fluorescence in the root segments under different Ca concentrations. (**A**), 0 – 5 mm; (**B**), 15 – 20 mm. Cd distribution along rice root visualized by staining with Leadmium Green AM dye. Cd exposure: 50 μM; Ca concentrations: 0.05, 0.5 and 5 mM. Data are means ±SE (n=6). Different letters represent the significant difference between treatments at $P<0.05$.

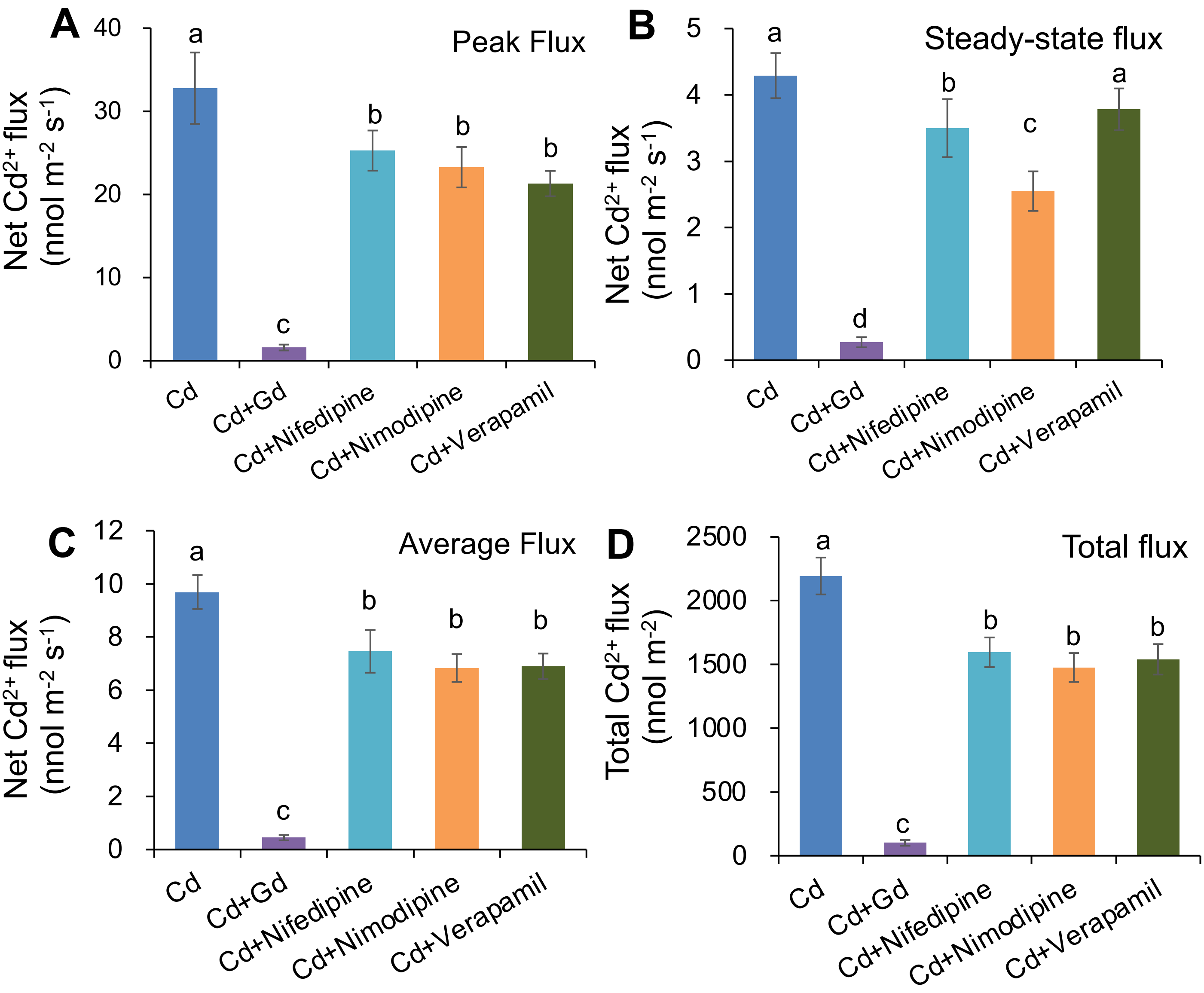

Figure S3. Effects of different Ca channel blockers on the characteristics of net Cd^{2+} flux from rice root epidermis. (**A**), Peak Cd^{2+} flux immediately after Cd treatment; (**B**), Steady-state Cd^{2+} flux at the end of the flux measurement; (**C**), Average Cd^{2+} flux over 25 min after Cd treatment; and (**D**), Total Cd^{2+} flux summed over 25 min after Cd treatment. Cd exposure: 50 μM; Ca channel blockers: 100 μM Gd^{3+}, NSCCs blocker; 20 μM nifedipine, DACCs blockers; 20 μM nimodipine, VDCCs blocker; 20 μM verapamil, HACCs blocker. Data are means ±SE (n=8). Different letters represent the significant difference between treatments at $P<0.05$.

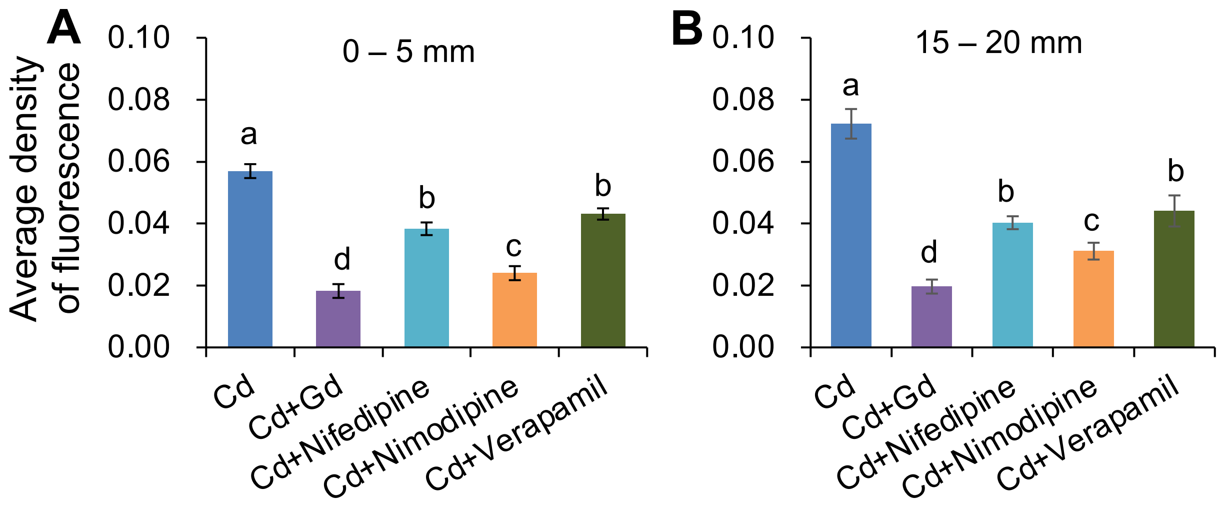

Figure S4. Average density of Cd green fluorescence in the root segments under different Ca concentrations. (**A**), 0 – 5 mm; (**B**), 15 – 20 mm. Cd distribution along rice root visualized by staining with Leadmium Green AM dye. Cd exposure: 50 µM; Ca channel blockers: 100 µM Gd^{3+}, NSCCs blocker; 20 µM nifedipine, DACCs blockers; 20 µM nimodipine, VDCCs blocker; 20 µM verapamil, HACCs blocker. Data are means ±SE (n=6). Different letters represent the significant difference between treatments at $P<0.05$.

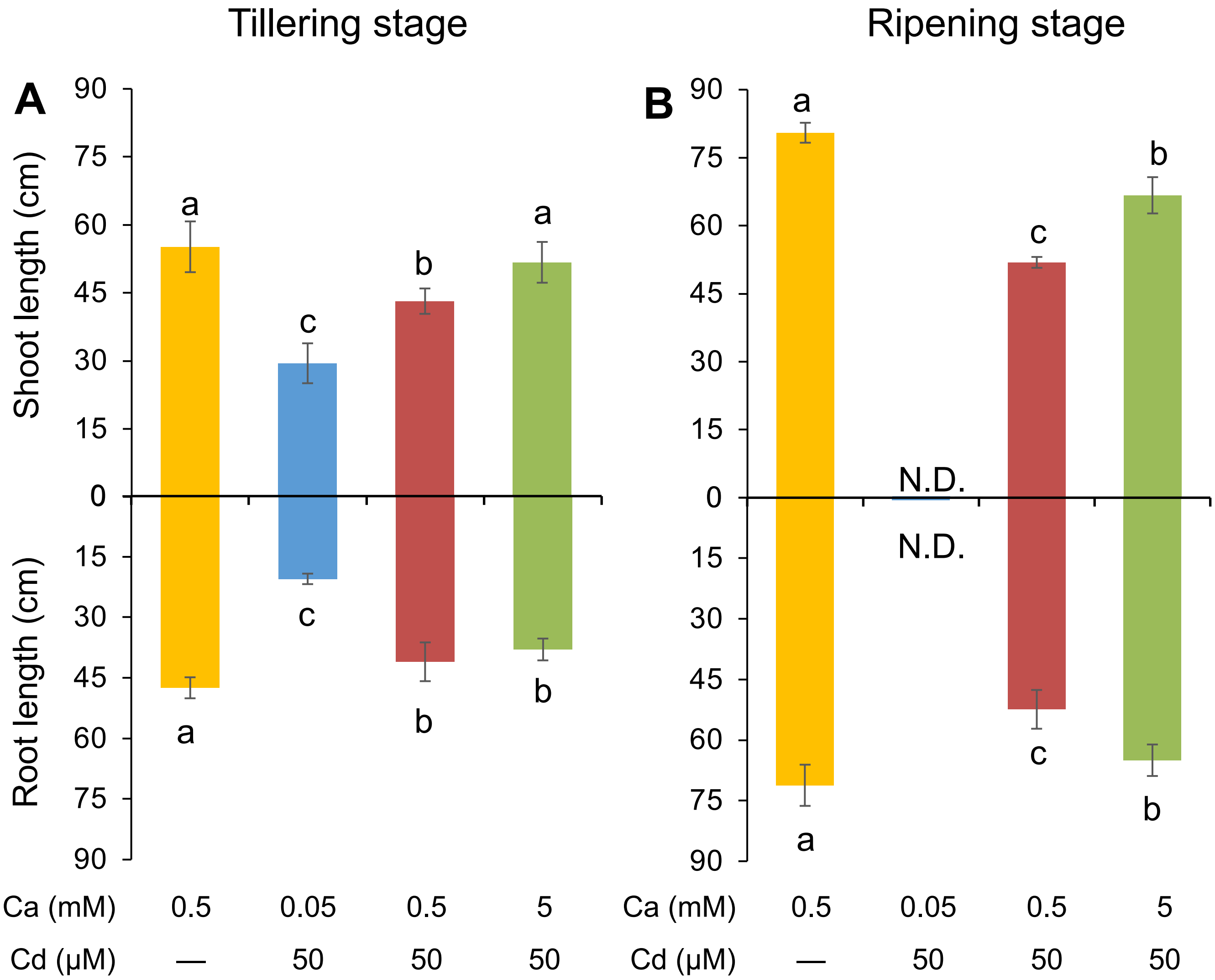

Figure S5. Effect of external Ca concentrations (0.05 mM, 0.5 mM and 5 mM) on the growth of rice plants with or without Cd stress. Shoot length and root length were determined at tillering stage (**A**) and ripening stage (**B**). Rice plants grown with 0.05 mM Ca + 50 μM Cd were died after one month of the treatment, so that data at ripening stage were not detected (N.D.). Different letters represent the significant difference between treatments at $P < 0.05$.

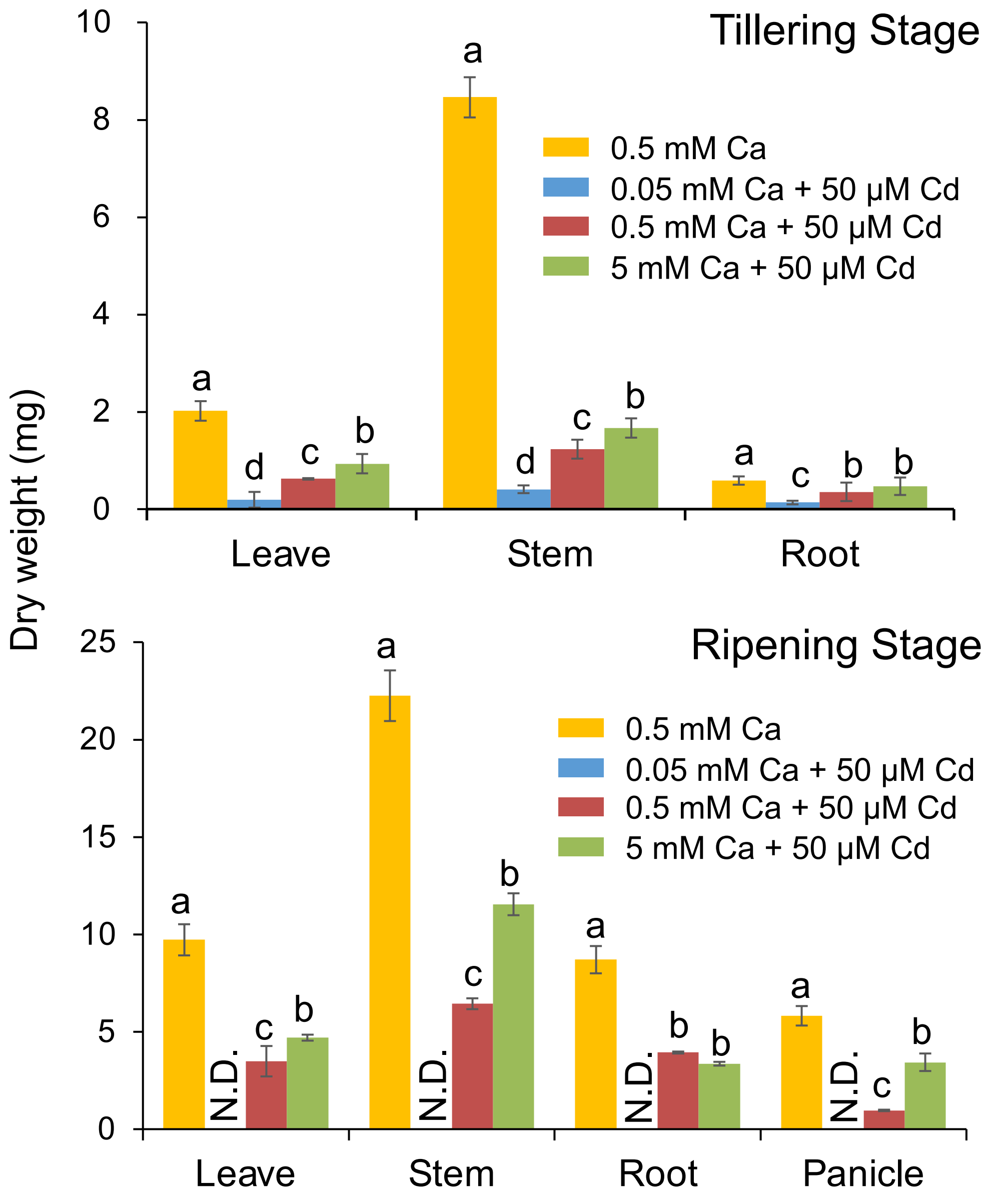

Figure S6. Effect of external Ca concentrations (0.05 mM, 0.5 mM and 5 mM) on biomass accumulation of rice plants with or without Cd (50 μM). Tissue dry weight were determined at tillering stage (**A**) and ripening stage (**B**). Rice plants grown with 0.05 mM Ca + 50 μM Cd were died after one month of the treatment, so that data at ripening stage were not detected (N.D.). Different letters represent the significant difference between treatments at $P < 0.05$.
