# Supplementary material for: Calcium Plays a Double-Edged Role in Modulating Cadmium Uptake and Translocation in Rice
Source: Int J Mol Sci. 2020 Oct 29;21(21):8058. doi: 10.3390/ijms21218058 (PMC7662230; doi:10.3390/ijms21218058)
Supplement: Supplementary file 1 [file ijms-21-08058-s001.zip › Supplementary Table S1 Primer sequences used for qRT-PCR.pdf]

Table S1 Primer sequences used for qRT-PCR

Gene name	Forward sequence5'-3'	Revers sequence5'-3'
OsNRAMP1	AGGAATACACCCGCATCAG	TACATACAGTGCCGGAGACA
OsNRAMP5	CGCTCCCAAGGTAGAGAAG AAGAA	ATATAAACGAACGGCTCCGAC GCA
OsHMA2	GGACGCATCGGACAATCAT	CATGTGCCAACATCTGCAA
OsHMA3	TCCATCCAACCAAACCCGG AAA	TGCCAATGTCCTTCTGTTCCC A
OsActin	GCATCTCTCAGCACATTCCA	GCGATAACAGCTCCTCTTGG
